# Novel Approaches for Imaging-Based Diagnosis of Ocular Surface Disease

**DOI:** 10.3390/diagnostics10080589

**Published:** 2020-08-13

**Authors:** Doreen Schmidl, Andreas Schlatter, Jacqueline Chua, Bingyao Tan, Gerhard Garhöfer, Leopold Schmetterer

**Affiliations:** 1 Department of Clinical Pharmacology, Medical University of Vienna, 1090 Vienna, Austria; doreen.schmidl@meduniwien.ac.at (D.S.); andreas.schlatter@meduniwien.ac.at (A.S.); gerhard.garhoefer@meduniwien.ac.at (G.G.); 2 Department of Ophthalmology, Vienna Institute for Research in Ocular Surgery-Karl Landsteiner Institute, Hanusch Hospital, 1140 Vienna, Austria; 3 Singapore Eye Research Institute, Singapore National Eye Centre, Singapore 169856, Singapore; jacqueline.chua.y.m@seri.com.sg (J.C.); bingyao.tan@ntu.edu.sg (B.T.); 4 Ophthalmology and Visual Sciences Academic Clinical Program, Duke-NUS Medical School, Singapore 169857, Singapore; 5 SERI-NTU Advanced Ocular Engineering (STANCE), Nanyang Technological University, Singapore 639798, Singapore; 6 Lee Kong Chian School of Medicine, Nanyang Technological University, Singapore 308232, Singapore; 7 Institute of Molecular and Clinical Ophthalmology, CH-4031 Basel, Switzerland; 8 Center for Medical Physics and Biomedical Engineering, Medical University of Vienna, 1090 Vienna, Austria

**Keywords:** ocular surface disease, dry eye, meibomian gland dysfunction, optical coherence tomography, in vivo confocal microscopy, meibography, interferometry, tear break-up time, ocular thermography, wavefront aberrometry

## Abstract

Imaging has become indispensable in the diagnosis and management of diseases in the posterior part of the eye. In recent years, imaging techniques for the anterior segment are also gaining importance and are nowadays routinely used in clinical practice. Ocular surface disease is often synonymous with dry eye disease, but also refers to other conditions of the ocular surface, such as Meibomian gland dysfunction or keratitis and conjunctivitis with different underlying causes, i.e., allergies or infections. Therefore, correct differential diagnosis and treatment of ocular surface diseases is crucial, for which imaging can be a helpful tool. A variety of imaging techniques have been introduced to study the ocular surface, such as anterior segment optical coherence tomography, in vivo confocal microscopy, or non-contact meibography. The present review provides an overview on how these techniques can be used in the diagnosis and management of ocular surface disease and compares them to clinical standard methods such as slit lamp examination or staining of the cornea or conjunctiva. Although being more cost-intensive in the short term, in the long term, the use of ocular imaging can lead to more individualized diagnoses and treatment decisions, which in turn are beneficial for affected patients as well as for the healthcare system. In addition, imaging is more objective and provides good documentation, leading to an improvement in patient follow-up and education.

## 1. Introduction

The ocular surface consists of the tear film, conjunctiva, cornea, eyelids and lacrimal glands. The term “ocular surface disease (OSD)” is frequently synonymous for dry eye disease (DED), which in its strict sense is not correct since DED is a condition that belongs to a group of ocular surface disorders, which also includes blepharitis, Meibomian gland dysfunction (MGD), allergic eye diseases and other conditions affecting the ocular surface [1].

Many diseases can cause ocular surface disorders. Table 1 provides an overview of clinical routine tests and new imaging techniques for OSD and shows parameters that are included in this review. We start this review paper with a summary of the most frequent forms of OSD where imaging may add clinical value in differential diagnosis and follow-up.

## 2. Ocular Surface Diseases for Imaging

### 2.1. Dry Eye Disease

Dry eye disease (DED), in the past often referred to as keratoconjunctivitis sicca, is defined as a multifactorial affection of the ocular surface. It is associated with a loss of homeostasis in the tear film and is accompanied by ocular symptoms. Tear film instability, hyperosmolarity, ocular surface inflammation and damage, as well as neurosensory abnormalities, are typical signs of the disease [2]. In clinical routine, diagnosis is usually based on slit lamp examination, tear film break-up time (TFBUT), Schirmer test score and corneal and conjunctival staining as well as measurement of tear osmolarity [3]. Common symptoms of DED include redness, burning, stinging, foreign body sensation, pruritus, photophobia and blurred vision ranging from mild to severe [3]. Symptoms can be systematically assessed and quantified by standardized questionnaires such as the Ocular Surface Disease Index (OSDI) or the Dry Eye Questionnaire (DEQ-5) [4].

Despite its worldwide prevalence ranging from 20 to 50% [5], diagnosis and monitoring of DED remain challenging in clinical practice [4]. A major issue relates to the weak correlation between clinical signs and symptoms in affected patients [6]. Another problem is the low reproducibility and the subjective nature of most assessments for DED [7]. Therefore, new imaging-based techniques may help to overcome these issues.

### 2.2. Meibomian Gland Dysfunction

Meibomian gland dysfunction (MGD), an umbrella term encompassing a variety of different meibomian gland disorders, is a common cause for DED. The International Workshop on Meibomian gland dysfunction defined MGD as ‘‘a chronic, diffuse abnormality of the meibomian glands, commonly characterized by terminal duct obstruction and/or qualitative/quantitative changes in the glandular secretion’’ [8]. The dysfunction of Meibomian glands impacts the meibum secreted and frequently affects the lipid layer of the tear film, leading to the evaporative form of DED. In addition, changes in eyelid morphology, altered secretions, gland dropout, Meibomian orifice plugging, hyperemia and telangiectasia can frequently be observed at the slit lamp examination as the most prominent clinical signs of the diseases. There is, however, a subgroup of patients who show no clinical signs but still suffer from MGD [9].

### 2.3. Conjunctivitis and Keratitis

Conjunctivitis and keratitis often come with DED, but can also have other causes, such as infections, traumas or allergies. Differential diagnosis from DED in these cases is essential, since signs and symptoms are often similar, but treatment is different [4].

## 3. Conventional Non-Imaging-Based Techniques

### 3.1. Slit Lamp Biomicroscopy

In clinical practice, OSD is usually diagnosed based on the slit lamp examination taken together with medical history and assessment of symptoms. Standard parameters for OSD to be evaluated during slit lamp examination without the need of dye instillation are conjunctival hyperemia, eyelid/Meibomian gland status, evaluation of debris and lid parallel conjunctival folds (LIPCOF) [4]. Using different dyes such as fluorescein or lissamine green, corneal epithelial defects and conjunctival staining can be made visible during slit lamp examination. Further, determination of tear break-up time using fluorescein, a frequently used parameter to assess tear film stability, plays a major role in diagnosis and follow up of OSD [4]. The major strengths of the slit lamp examination are the global availability in ophthalmologic and optometrists’ practices and the low costs. However, it needs to be considered that the evaluations are subjective and that there is no objective documentation unless slit lamp photographs or videos are taken. As mentioned above, since for some examinations instillation of dyes is necessary, they cannot be considered as truly non-invasive and the dye insertion may have an impact on the outcomes by interfering with tear film homeostasis [4].

### 3.2. Impression Cytology

Impression cytology allows for evaluation of cell morphology. Briefly, a filter is pressed towards the conjunctiva and via different staining methods cell morphology can then be assessed under the microscope. A common parameter evaluated by the means of impression cytology is assessment of squamous metaplasia, reflected in the reduction of goblet cells and alterations of epithelial cells. Inflammatory cells can also sometimes be observed. In addition, several biomarkers can be assessed using immunofluorescence staining, flow cytometry, multiplex bead immunoassays or real-time PCR. Impression cytology can be applied in various diseases of the ocular surface because it provides information about conjunctival histology. The technique, however, requires that a filter is pressed against the conjunctiva and therefore cannot be considered to be non-invasive [10].

## 4. Imaging Modalities

### 4.1. Anterior Segment Optical Coherence Tomography

Over the past 20 years, OCT has revolutionized ocular imaging and clinical care of patients with retinal or optic nerve disease. Diagnosis, as well as follow-up, is nowadays inconceivable without this technique. Continuous improvement in acquisition speed, sensitivity, field of view and axial resolution has played a major role in this respect [11]. OCT has been listed by the International Dry Eye Workshop as a potential diagnostic tool for DED and indeed, it can be used for several relevant assessments [4].

Briefly, OCT measures light backscattered from tissue as a function of the echo time delay, thereby creating 2- or 3-dimensional tomographic images. It consists of a low-coherence light source, where the emitted light is split into two paths by a beam splitter. These two paths then enter two arms, a reference arm reflecting light from a mirror and a sample arm where the light is scattered by the tissue under investigation. The reflected and backscattered light interferes at the beam splitter and the resulting fringes are captured by a detector. From this information, an image can be created with micrometer axial resolution [11]. OCT systems for the anterior segment are capable of obtaining images of important structures of the ocular surface. While commercially available systems typically feature axial resolutions of approximately 5 μm, custom-built ultrahigh-resolution systems with a broadband light source offer axial resolutions in the order of 1 μm [11,12,13,14,15]. The lateral resolution is given by the numerical aperture of the focusing optics and central wavelength, and typically ranges from 15 to 60 μm [11]. Recently, an in-depth review about anterior segment OCT was published to which the readers can refer for more technical details [11].

**Figure 1:**
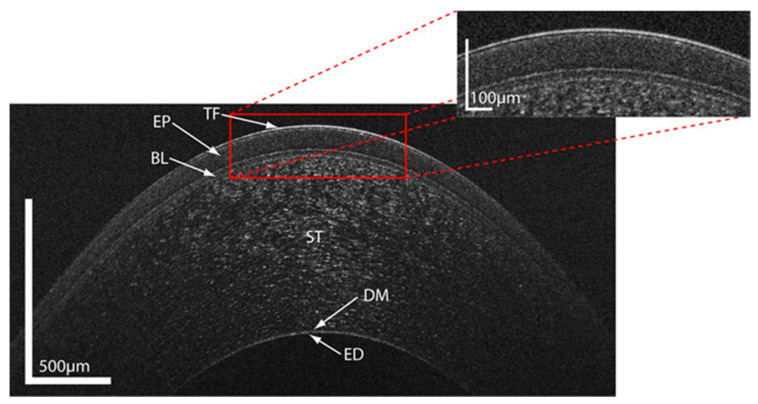
Ultrahigh resolution optical coherence tomography image of the healthy human cornea. TF = tear film; EP = corneal epithelium; BL = Bowman’s layer; ST = corneal stroma; DM = Descemet’s membrane; ED = corneal endothelium. The scales for the *x*- and *y*-axis are indicated. Reprinted from Werkmeister et al., 2013 [12].

As shown in Figure 1, all layers of the cornea and the precorneal tear film can be visualized [12].

A high resolution OCT system can visualize small corneal epithelial defects (superficial punctate keratopathy), which are frequently found in patients with OSD [16]. Larger corneal abrasions can be made visible and quantified, which allows for non-invasive monitoring of wound healing [17]. Therefore, OCT is an alternative for the clinical assessment of epithelial defects without the need for the use of dyes.

OCT of the cornea has also been used as a tool in diagnosis and follow-up of microbial infectious disease, such as fungal [18], viral [19], bacterial [20] and parasitic keratitis [21] that show characteristic features on images, but no quantitative parameters for this purpose have yet been established.

As the tear-film itself is the most crucial part in DED, the non-invasive imaging of the tear film by OCT has gained popularity in the last few years and several approaches have been realized. As such, pre-corneal tear film thickness (TFT) can be measured with ultrahigh resolution OCT systems [12,22,23] and TFT has been found to be a new parameter in OSD that correlates with objective and subjective assessments, such as tear film break-up time (TFBUT) and OSDI [24]. Studies assessing TFT in both patients with DED and healthy subjects reported that patients with dry eye show lower TFT compared to healthy controls, who have an average TFT of approx. 5 μm as measured using OCT [24,25]. These values of TFT are in keeping with values as reported using an alternative interferometric method [26,27]. TFT can also be used as a surrogate to determine the duration of the effect of lubricating eye drops on the tear film since single administration leads to an increase over a certain amount of time [25,28,29,30]. In contrast, instillation of low viscosity agents such as physiological saline have almost no effect on TFT and therefore can be used as a control in studies [28,30]. This has been observed in both healthy subjects as well as in patients with DED with different eye drops [25,28,29,30]. Whereas the effect of most lubricants on TFT is relatively short in time, ranging between several minutes and a few hours, some agents have been shown to increase TFT up to 24 h, such as topical chitosan-N-acetylcysteine, perfluorohexyloctane or low dose hydrocortisone [31,32,33].

While TFT refers to the overall pre-corneal tear film, including the lipid, aqueous and the mucous layer, there have also been some approaches to measure the thickness of specific tear film layers by OCT separately such as the lipid layer and the aqueous layer [34,35,36]. Currently, studies using these approaches for therapy evaluation, or to investigate patients with different forms of OSD, are still lacking.

**Figure 2:**
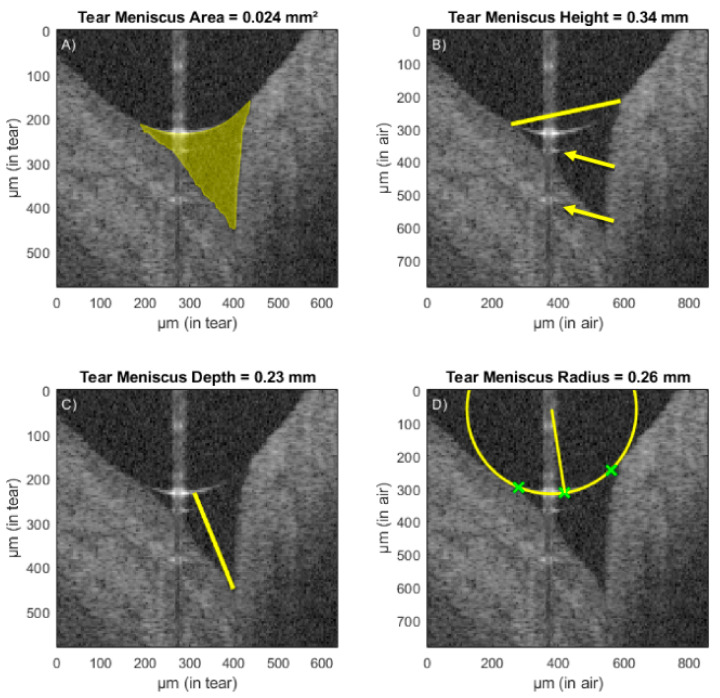
Assessment of tear meniscus area (**A**), tear meniscus height (**B**), tear meniscus depth (**C**) and tear meniscus radius (**D**) using ultrahigh-resolution optical coherence tomography. Reprinted from Stegmann et al. 2019 [36].

Evaluation of the tear meniscus with OCT has been proposed as another parameter for studying OSD (Figure 2) [11]. The tear meniscus is the accumulation of tear fluid between the eyelid margin and the ocular surface, containing 75–90% of the tears [37,38]. While methods for measurement of tear meniscus height at the slit lamp have been introduced previously, evaluation of this parameter using OCT offers several advantages omitting reflex tearing due to bright light and providing good repeatability [39,40]. In addition, not only the height of tear meniscus, but also the area, volume, depth and radius can be measured (Figure 2) [36].

Tear meniscus parameters correlate negatively with OSDI and corneal fluorescein staining and positively with Schirmer scores in patients with DED [41,42,43,44]. In addition, tear meniscus parameters differ between different types of dry eye and may therefore be valuable in differential diagnosis [45]. Single administration of topical lubricants increases tear meniscus parameters in healthy subjects as well as in patients with OSD and may therefore also be used as a marker for corneal residence time of eye drops [46,47,48]. Two-month treatment with topical cyclosporine increased tear meniscus height and volume in patients with dry eye, suggesting that this parameter might also be valuable in therapeutic monitoring [13].

Appearance of lid-parallel conjunctival folds (LIPCOFs) is frequently seen in DED and is usually recognized at the slit lamp. Recently, imaging of LIPCOFs has been reported using OCT, which offers the advantage of providing greater details of LIPCOF morphology and area [49]. In addition, this parameter has been found to correlate with slit lamp grading and the Dry Eye Questionnaire (DEQ) [49,50].

Another structure of interest in OSD is the lacrimal drainage system, in particular the lacrimal punctum and the canaliculi. In patients complaining about epiphora, obstruction of the lacrimal drainage system needs to be excluded. This is usually done invasively via probing [51]. Punctuae and canaliculi can, however, also be identified by OCT [52,53,54]. This approach is non-invasive and provides punctual dimensions quantitatively, therefore allowing for evaluation of therapeutic success without the need for probing [53].

OCT has also been used for Meibomian gland imaging using custom-built, as well as commercially available, devices [55,56,57]. Using Optical Coherence Tomography Meibography (OCT-M) a significant decrease in Meibomian gland length and width was observed in patients with Meibomian gland dysfunction (MGD) [58].

### 4.2. In Vivo Confocal Microscopy

In vivo confocal microscopy (IVCM) offers the opportunity to extract an en-face microstructure image at the cellular level in real time [59,60]. In comparison to OCT, it provides a higher lateral resolution of approximately 1.0 µm but with a significantly smaller field of view [59]. Although it is possible to image all corneal layers, the focus can only be set to one plane of interest at a time and different corneal layers have to be imaged separately [59,60]. Another disadvantage is that the examination requires direct contact with the eye, therefore inducing discomfort for the patient and carrying a small risk of corneal epithelial defects [59].

Several structures of the ocular surface can be studied using IVCM. In clinical practice, IVCM has gained importance in the management of corneal diseases, especially atypical infections, which are often sight-threatening but are difficult to diagnose. As such, several studies report on the usefulness of IVCM in the differential diagnosis of acanthamoeba, fungal, bacterial and viral keratitis [59,61]. IVCM has also been used to monitor corneal wound healing, although it cannot directly measure defect size, since the field of view is too small, but injured corneal epithelial cells can be visualized and characterized according to morphology, and the time until a healthy epithelium is re-established can be measured [62].

While it is not possible to directly visualize small epithelial defects as with fluorescein staining or OCT, it is possible to use epithelial cell density as a surrogate parameter for corneal epithelial damage in OSD [63]. Indeed, corneal epithelial cell density increased in parallel with improvement in corneal fluorescein staining in a longitudinal study in patients with DED receiving topical cyclosporine [64].

**Figure 3:**
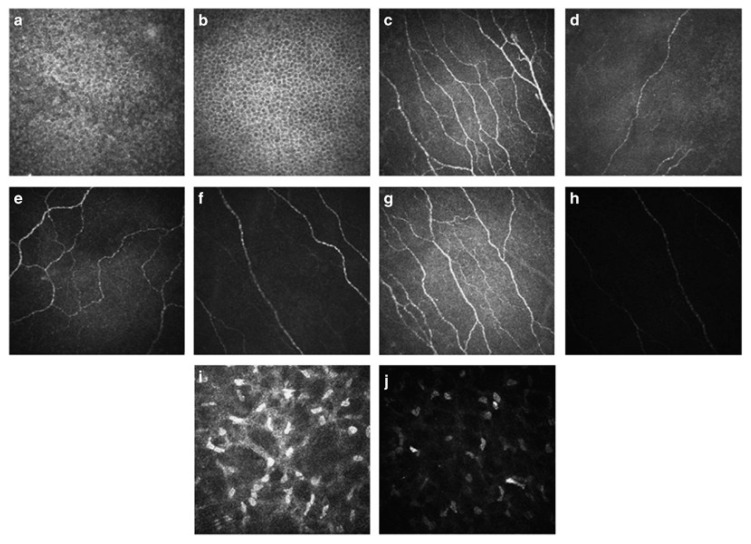
IVCM images of a patient with dry eye disease before and after six months of treatment with topical cyclosporine. Cell density of the intermediate epithelium of the cornea (**a**,**b**), number of nerve fibers of subbasal plexus in the cornea (**c**,**d**), nerve fiber tortuosity of subbasal plexus in the cornea (**e**,**f**), nerve fiber reflectivity of subbasal plexus in the cornea (**g**,**h**) and keratocyte activation in the cornea (**i**,**j**) are shown, the first picture always represents baseline and the second picture findings after six months therapy in the same patient. Reprinted from Iaccheri et al., 2017 [64].

OSD is often associated with loss and damage of corneal nerves, which partially explains the weak correlation between signs and symptoms, since a reduction in corneal sensory nerves leads to a reduced pain perception [65]. While previous investigation of corneal nerves was only possible by in vitro methods, such as light or electron microscopy with staining, IVCM offers the possibility to gain information about corneal nerves in vivo [66]. IVCM corneal nerve parameters include the number of nerves per frame reported as the number of nerves/mm^2^, nerve density in mm/mm^2^, number of beadings per 100 μm, nerve tortuosity and reflectivity (both assessed on a grading scale) and nerve length and width in µm [63,67]. These corneal nerve parameters correlated significantly with OSDI in a previous study [67]. In patients with DED as well as in patients on long-term topical glaucoma therapy with OSD symptoms, a reduction in corneal nerve number and density was found, which significantly correlated with corneal sensitivity [68]. Treatment with topical cyclosporine increased corneal nerve number and density, making these parameters an interesting surrogate for OSD, especially in more severe cases (Figure 3) [69].

In addition to the cornea, the conjunctiva can be investigated using IVCM: Goblet cell density can be measured by IVCM, which was in the past only possible by invasive technologies such as impression cytology, requiring time-consuming staining procedures [70,71]. Inflammatory cells, which are often found in patients with OSD, can also be visualized using IVCM. This is again an attractive alternative to using impression cytology with the same disadvantages as described above [72,73].

IVCM also offers the possibility to image the microstructure of Meibomian glands [74]. Based on images obtained with IVCM, Meibomian glands can be graded similar to the meibography score and a strong correlation was found between the two approaches [74]. In a cross-sectional study in DED patients, dry eye symptoms correlated negatively with parameters for MGD severity as obtained by MGD [75]. In summary, IVCM is a valuable tool in the diagnosis and management of MGD, but its need for contact with the ocular surface prevents application in large patient populations.

### 4.3. Non-Contact Meibography

Meibography refers to the visualization and quantification of Meibomian gland drop-out using photodocumentation [76] and offers observation of the silhouette of the Meibomian gland morphological structure by transillumination of the everted eyelids [4]. Conventional meibography was introduced in the 1970s and although it was possible to visualize Meibomian gland structure in vivo, the associated discomfort for the patient prevented widespread use [77]. Briefly, the everted eyelid had to be transilluminated with a probe consisting of a sharp tip and a bright light, thereby producing heat [78].

**Figure 4:**
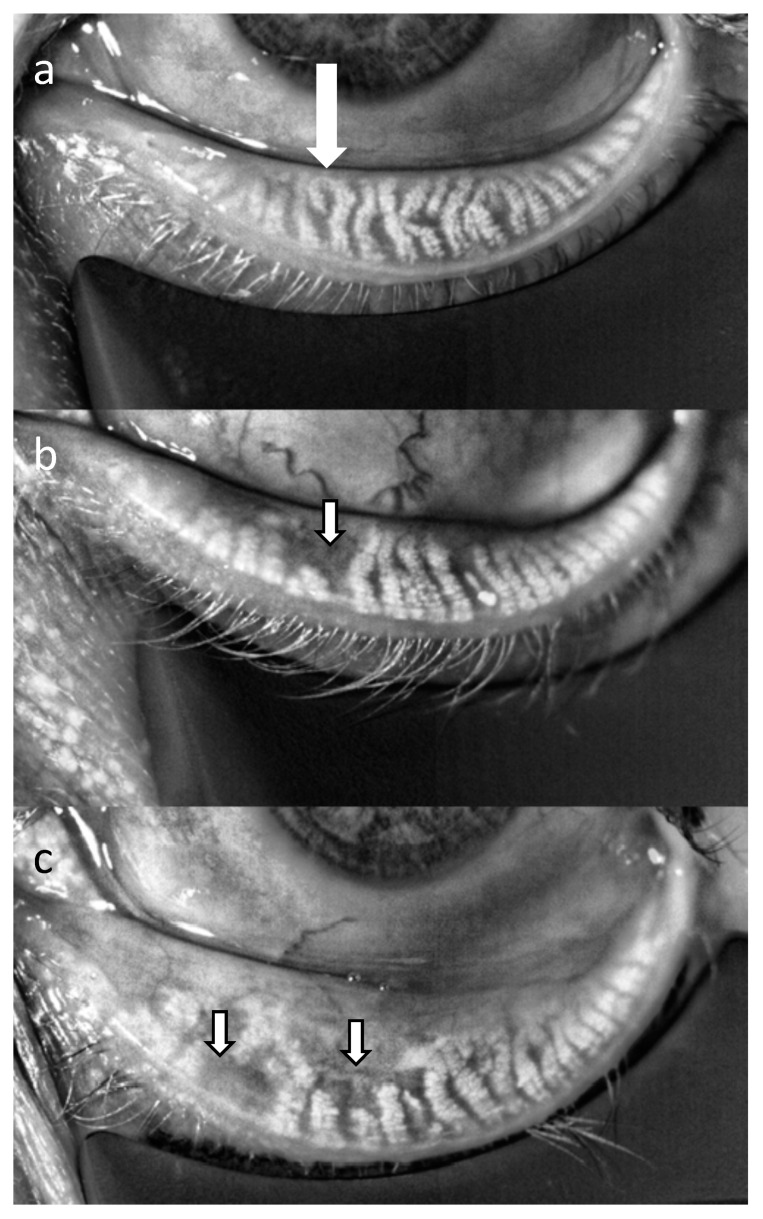
Infrared images of the Meibomian glands of the lower eyelid in a healthy subject (**a**), a patient with mild Meibomian gland dysfunction (MGD) (**b**) and a patient with moderate MGD (**c**). Areas of gland dropouts can be clearly seen (arrows). The images were taken with the LipiView interferometer (TearScience, Morrisville, NC, USA).

To overcome these disadvantages, systems for non-invasive imaging of Meibomian glands have been developed. Non-contact meibography is based on the autofluorescence of healthy meibum when illuminated with infrared light which can be detected by an infrared charge-coupled device camera [77,79]. Loss of glands, lack of meibum or alterations in meibum composition are seen as dark lesions with this technology (Figure 4) [77,79]. From obtained images or videos, Meibomian gland loss can be quantified as a diagnostic parameter, called Meiboscore [80]. Currently, slit lamp based, mobile and topography-equipped systems are commercially available for this purpose [77]. In a cross-sectional retrospective analysis, Meiboscore correlated significantly with expressible Meibomian glands and TFBUT [80]. In a prospective study, Meiboscore was significantly higher in patients with posterior blepharitis compared to healthy controls [81]. Meibography has also been used to evaluate therapeutic success of treatment with topical diquafosol, a P2Y2 purinergic receptor agonist, or probing of Meibomian glands in patients with obstructive MGD [82,83]. Investigating side effects of drugs such as antiglaucoma medications or isotretinoin on Meibomian glands is another application of the technique [84,85]. Newer systems allow real-time monitoring with meibography videos [86].

### 4.4. Interferometry

**Figure 5:**
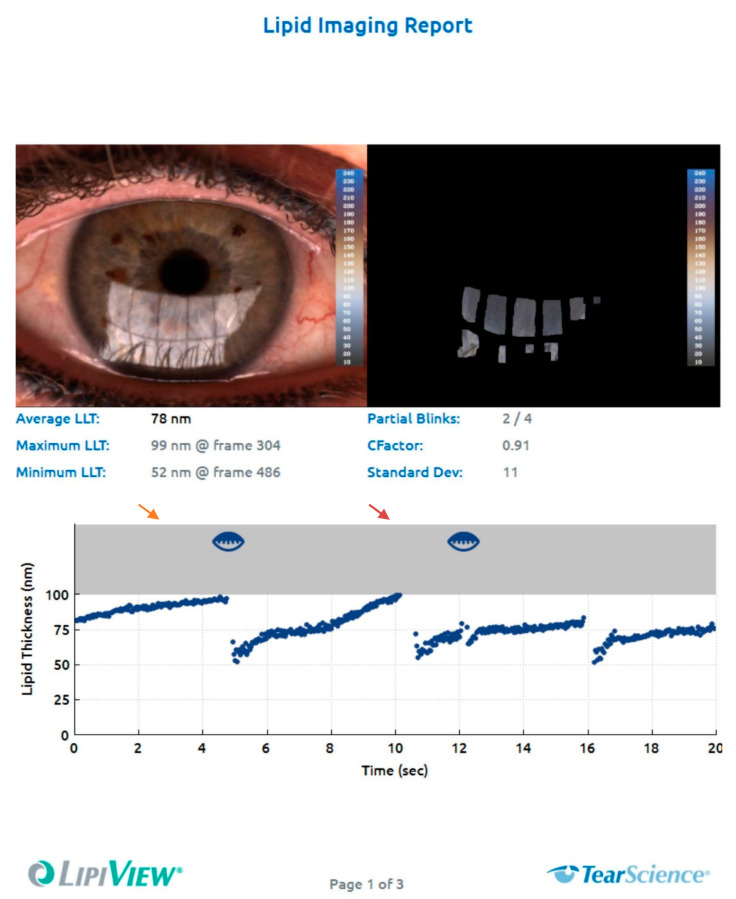
Measurement of lipid layer thickness and blink frequency with the LipiView Interferometer (TearScience, Morrisville, NC, USA). The system provides average, maximum and minimum lipid layer thickness (LLT) values in absolute nm values, measured over a period of 20 s. In addition, blink frequency is registered, including complete and partial blinks. The arrows indicate partial blinks.

Imaging-based ocular surface interferometry allows the measurement of lipid layer thickness (LLT) on a nanometer (nm) scale [87]. Briefly, the ocular surface is illuminated with a white light-emitting diode (LED) light source and the resulting image is recorded using a CCD camera. Optical interference colors on the surface of the tear film can then be correlated to specific thickness values of the lipid layer (Figure 5) [87]. This is a major advantage compared to previously used techniques for the assessment of the lipid layer, which were also based on interferometry, but only provided qualitative grading at the slit lamp [88,89].

Patients with obstructive MGD have significantly thinner LLT than healthy subjects [90], whereas hypersecretory MGD results in an increase in LLT [91]. The lipid layer was also altered in patients with allergic conjunctivitis [92].

Studies investigating the short-time effect of single instillation of eye drops on LLT have been performed. While some studies report an immediate increase in LLT after instillation [93,94,95,96], other studies report no short-time effects of eye drops targeting the lipid layer of the tear film [28]. This could be due to technical limitations, because the natural lipid layer and the ingredients of the eye drops have different refractive indices, which affect the correct assessment of the LLT by interferometry [28,31]. Alternatively, the effect on the LLT may build up after multiple instillations [28]. Therefore, LLT may be used to monitor the effect of treatment on the lipid layer over time. Indeed some studies have observed an improvement of lipid layer thickness after a few weeks of instillation using lubricants as compared to placebo [31,97].

Eyes receiving glaucoma therapy or after undergoing cataract surgery show a significantly reduced LLT [98,99]. LLT may therefore also be used to monitor the detrimental effects of drugs or surgical procedures on the ocular surface.

Interferometry has also been used to measure lower tear meniscus height [100]. Tear meniscus height values measured with the DR-1α tear interferometer (Kowa, Tokyo, Japan) significantly correlated with values obtained from anterior segment OCT and were found to be significantly higher in healthy subjects compared to patients with aqueous deficient dry eye [100].

### 4.5. Non-Invasive Tear Film Break-Up Time

In clinical routine, tear film break-up time (TFBUT) is assessed at the slit lamp and is dependent on the amount of fluorescein solution instilled in the conjunctival fornix, thereby featuring suboptimal accuracy and repeatability in DED [101]. Hence, non-invasive tear film break-up time (NITFBUT), where no fluorescein instillation is required, has been proposed as an alternative to assess tear film stability [4]. Commercially available corneal topography systems can measure NITFBUT through the observation of placido disk images over time, which are reflected from the anterior surface of the eye [4,102]. Using specifically developed software, these corneal topography systems are able to assess localized changes in corneal power and break up of the tear film [103,104,105,106]. NITFBUT can also be assessed with specific keratography modalities [107,108]. High-speed videokeratoscopy detects changes in the variance of stability in image quality, by the variance of the number of complete rings of the placido disk detected radially from the center [109,110]. This instability of the tear film is used as an estimate of NITFBUT.

Different technical approaches to measure NITFBUT used in several studies indicate sensitivity values between 82–84% and specificity values between 76–94% for detecting dry eye [108,111,112]. NITFBUT was found to correlate with TFBUT as assessed at the slit lamp, as well as with Schirmer test, tear osmolarity and OSDI [113,114].

NITFBUT values of DED patients have been reported to be on average only 25% to 32% of normal values assessed in healthy subjects. Values of less than 10 s are usually considered as DED when viewing the reflection of an illuminated grid pattern [112]. Therapy with topical lubricants improves NITFBUT in a similar fashion to TFBUT [31,115].

### 4.6. Ocular Thermography

Thermography measures the surface temperature of an object non-invasively using a thermographic camera operating within the infrared range. Technically speaking, the higher the temperature of the surface, the higher the power per radiating area. For the human body, this radiation is several hundred Watts in the infrared range [116]. Initially, thermography was invented to measure changes in skin temperature. It was then further developed to detect changes in ocular surface temperature and accuracy; resolution and speed of the technique have increased continuously [117,118,119,120,121].

Since inflammation is usually associated with elevated temperature, it comes as no surprise that in ocular inflammatory diseases such as scleritis or anterior uveitis, ocular surface temperature in the affected eye is higher than in the contralateral non-affected eye [122].

This also holds true for DED, which also comes with inflammation, and indeed patients with DED featured higher temperatures of the ocular surface than healthy controls [123]. However, probably due to a greater rate of tear film evaporation, patients with DED also show a faster cooling rate (loss of heat) than healthy subjects [118,121,124].

A greater variance of temperature across the ocular surface as well as fluctuation in temperature over a short period of time (a few seconds after blinking) has been found in patients with DED [125]. In addition, these parameters seem to be associated with lower TFBUT in these patients [123,126]. Ocular surface temperature variation and TFBUT in different areas of the cornea were correlated in some studies [127,128]. A correlation between tear meniscus height, Schirmer test score and ocular surface temperature difference has also been reported [126].

Based on these findings, thermography has been applied to differentiate between DED of differing etiologies. Aqueous deficient dry eye patients seem to have lower temperatures of the ocular surface but more substantial heat loss during the interblink intervals, whereas evaporative dry eye patients seem to show lower cooling rates [129,130].

### 4.7. Tear Film Imager

The tear film imager (TFI), developed by AdOM (Advanced Optical Technologies Ltd., Lod, Israel) can directly and dynamically measure tear physiology with one measurement. Using spectral interference technology, it offers a high lateral resolution in the nanometer range and provides images of the corneal surface of a large field of view of 6 mm diameter [131] tolerating the natural eye state of blinks, saccadic motions, and defocusing events [132]. The TFI allows measurements of the muco-aqueous layer thickness (MALT) [133] and the lipid layer thickness (LLT) [131].

Momentary drops in the lipid layer thickness can be detected by the TFI, which defines the lipid break-up time (LBUT). The LBUT values as measured by the TFI significantly correlated with the clinical measurements of TBUT while MALT values significantly correlated with Schirmer score [131]. In particular, TFI data shows thinner MALT in patients with DED compared to healthy subjects [131]. According to the clinical DED categorization, the TFI features a sensitivity of 87% and a specificity of 88% [133]. However, currently only a few studies implementing this device in OSD research are available.

### 4.8. Blink Pattern Analysis

Low blink rate as well as incomplete blinking are well-recognized risk factors for OSD [134]. Devices to investigate blink patterns have therefore been developed based on a video camera recording of blinks. The devices are capable of identifying incomplete blinks during a specified amount of time (Figure 5) [135] and the number of incomplete blinks has been found to negatively correlate with the sum of both TFBUT and LIPCOF [136,137]. In addition, it has been shown that DED patients have prolonged eyelid-closed time and shorter blink intervals, which again correlates with TFBUT [138,139,140].

An investigation of blink patterns is of interest for the assessment of smart glasses during visual tasks such as reading [141]. Patients with moderate DED show a reduced spontaneous eye-blink rate during video display terminal use, which results in a worsening of DED symptoms [142].

### 4.9. Wavefront Aberrometry

Wavefront aberrometry is commonly used in preparation for refractive surgery. Briefly, aberrometry uses wavefront sensing, where a light wave is sent into the eye and aberrations in the returning waves correspond to changes in refraction. While wavefront aberrations caused by ametropy, astigmatism, corneal disorders such as keratoconus or changes in the lens remain stable when sequential measures are applied, changes in the tear film lead to fluctuations in the dynamic wavefront analysis. This offers the possibility to analyze visual performance related to tear film dynamics [143].

Indeed, wavefront aberrations occur earlier in patients with DED compared to healthy controls when measured over approximately 30 s, which is probably caused by instability of the tear film. Furthermore, they correlate with tear meniscus parameters as well as with OSDI [144,145]. In a study using a driving simulator, the response time of DED patients was positively correlated with progression index for higher-order aberrations (HOA) over 10 s [146]. One study investigated the effect of single instillation of topical lubricants on dynamic wavefront aberrometry in patients with DED, but no changes were found [147]. In contrast, another clinical trial investigated the effect of rebamipide ophthalmic suspension, a secretagogue, on dynamic wavefront aberrometry in patients with DED. After four weeks of treatment, a significant stabilization in the short-time course of corneal HOAs was found [148]. Studies on the effect of long-term treatment of OSD on dynamic wavefront aberrometry are currently lacking.

## 5. Conclusions and Perspective

In ophthalmology, imaging technologies are gaining importance and are becoming a significant part of the clinical routine for a wide variety of conditions. While for the posterior segment, OCT has become the gold-standard in the diagnosis and follow-up of many diseases such as age-related macular degeneration [149], diabetic retinopathy [150] and glaucoma [151], imaging techniques for the ocular surface are not well established. Although the techniques introduced in this review represent promising approaches, they are still more cost intensive than clinical standard tests at the slit lamp such as determination of TBUT or corneal fluorescein staining. On the other hand, OSD is becoming an increasing economic burden and more specific diagnosis and management would be beneficial and more cost-efficient in the long term [152]. Imaging provides improved documentation and objective assessment, which is particularly beneficial in the long-term follow up and can also be used for patient education. These advantages have to be weighed against the financial requirements for the systems. Centers that are specialized in the clinical care of dry eye cases would definitely benefit from the use of imaging systems. All the techniques introduced provide different parameters and therefore not one single approach will fit all clinical needs. Concrete recommendations on which system to use is difficult, because there is a lack of cost-effectiveness models in the literature. Cost effectiveness may also largely depend on the clinical setting. In some countries dry eye patients are mainly seen by optometrists, whereas in other countries clinical care is mainly provided by ophthalmologists. In centers where OCT systems are available, their application to dry eye disease may be a reasonable approach, but commercial software for this use is currently not well developed. Another problem with anterior segment OCT is that the axial resolution in commercially available instruments is still too low for assessment of tear film parameters. Given the high prevalence of the disease, cost effectiveness models for different clinical scenarios are urgently required.

Another important field related to imaging is the application of artificial intelligence [153]. Deep learning algorithms have been applied in several image-centered medical specialties such as radiology, pathology, dermatology and also ophthalmology. Using digital fundus images, diabetic retinopathy, glaucoma, age-related macular degeneration, retinopathy of prematurity and refractive error can be detected by deep learning systems [153]. One approach for the objective evaluation of OSD, in particular allergic conjunctival disease, has been introduced recently [154]. In addition, convolutional networks have been developed for segmentation of different structures in anterior segment images [155,156]. In the future, algorithms can potentially also be applied to various forms of OSD, with the objective images captured by the techniques that have been introduced in the present review. Using these images in addition to other clinical variables and reference standards, datasets can be created to develop convolutional neural networks for detection of different OSD subtypes. Deep learning approaches may also be used to identify patients that benefit from different treatments to allow for individualized medicine in this field. For this, however, large datasets will be required from international collaborations and multicenter trials.

## 6. Method of Literature Search

For this narrative review, a literature search was performed using PubMed. We included review articles and clinical trials relevant to the subject of the review, while case reports, case series and articles not written in the English language were excluded. Since the aim of the review was to give an oversight on current techniques for ocular surface imaging, the following search terms were used in various and/or logic combinations: “ocular surface disease”, “dry eye”, “Meibomian gland dysfunction”, “conjunctivitis”, “keratitis”, “tear film”, “optical coherence tomography”, “in vivo confocal microscopy”, “meibography”, “interferometry”, “non-invasive tear break up time”, “ocular thermography”, “tear film imager”, “blink pattern analysis”, “wavefront aberrometry”, “artificial intelligence” and “deep learning”.

## Figures and Tables

**Table 1:** The lines list the techniques that are covered in this review, the columns list the different parameters that can be assessed. LIPCOF = lid parallel conjunctival folds, OCT = Optical coherence tomography, IVCM = in vivo confocal microscopy, NITFBUT = non-invasive tear film break-up time. * requires instillation of fluorescein.

Technique	Slit Lamp Bio-Microscopy	Impression Cytology	OCT	IVCM	Non-Contact Meibography	Interferometry	NITFBUT	Ocular Thermography	Tear Film Imager	Blink Pattern Analysis	Wavefront Aberrometry
Parameter											
Superficial Punctuate Keratopathy	X *		X								
Corneal Epithelial Defects	X *		X	X							
Corneal Nerves				X							
Tear Film Thickness			X								
Tear Meniscus Height	X		X			X					
Lipid Layer Thickness			X			X			X		
Muco-Aqueous Layer Thickness									X		
Tear Film Break-Up Time	X *						X				
LIPCOF	X		X								
Goblet Cell Count		X		X							
Inflammatory Cells		X		X							
Meibomian Gland Evaluation	X		X	X	X						
Ocular Surface Temperature								X			
Optical Properties											X
Blink Pattern										X	

## References

[1] Khanna R.C. (2017). Ocular surface disorders. Community Eye Health.

[2] Craig J.P., Nichols K.K., Akpek E.K., Caffery B., Dua H.S., Joo C.K., Liu Z., Nelson J.D., Nichols J.J., Tsubota K. (2017). TFOS DEWS II Definition and Classification Report. Ocul. Surf..

[3] Messmer E.M. (2015). The pathophysiology, diagnosis, and treatment of dry eye disease. Dtsch. Arztebl. Int..

[4] Wolffsohn J.S., Arita R., Chalmers R., Djalilian A., Dogru M., Dumbleton K., Gupta P.K., Karpecki P., Lazreg S., Pult H. (2017). TFOS DEWS II Diagnostic Methodology report. Ocul Surf..

[5] Stapleton F., Alves M., Bunya V.Y., Jalbert I., Lekhanont K., Malet F., Na K.S., Schaumberg D., Uchino M., Vehof J. (2017). TFOS DEWS II Epidemiology Report. Ocul. Surf..

[6] Begley C.G., Chalmers R.L., Abetz L., Venkataraman K., Mertzanis P., Caffery B.A., Snyder C., Edrington T., Nelson D., Simpson T. (2003). The relationship between habitual patient-reported symptoms and clinical signs among patients with dry eye of varying severity. Investig. Ophthalmol. Vis. Sci..

[7] Zeev M.S., Miller D.D., Latkany R. (2014). Diagnosis of dry eye disease and emerging technologies. Clin. Ophthalmol..

[8] Nichols K.K., Foulks G.N., Bron A.J., Glasgow B.J., Dogru M., Tsubota K., Lemp M.A., Sullivan D.A. (2011). The international workshop on meibomian gland dysfunction: Executive summary. Investig. Ophthalmol. Vis. Sci..

[9] Chhadva P., Goldhardt R., Galor A. (2017). Meibomian Gland Disease: The Role of Gland Dysfunction in Dry Eye Disease. Ophthalmology.

[10] Hagan S. (2017). Biomarkers of ocular surface disease using impression cytology. Biomark Med..

[11] Ang M., Baskaran M., Werkmeister R.M., Chua J., Schmidl D., Aranha Dos Santos V., Garhöfer G., Mehta J.S., Schmetterer L. (2018). Anterior segment optical coherence tomography. Prog. Retin. Eye Res..

[12] Werkmeister R.M., Alex A., Kaya S., Unterhuber A., Hofer B., Riedl J., Bronhagl M., Vietauer M., Schmidl D., Schmoll T. (2013). Measurement of tear film thickness using ultrahigh-resolution optical coherence tomography. Investig. Ophthalmol. Vis. Sci..

[13] Wang J., Cui L., Shen M., Perez V.L., Wang M.R. (2012). Ultra-high resolution optical coherence tomography for monitoring tear meniscus volume in dry eye after topical cyclosporine treatment. Clin. Ophthalmol..

[14] Huang J., Yuan Q., Zhang B., Xu K., Tankam P., Clarkson E., Kupinski M.A., Hindman H.B., Aquavella J.V., Suleski T.J. (2014). Measurement of a multi-layered tear film phantom using optical coherence tomography and statistical decision theory. Biomed. Opt. Express.

[15] Bai Y., Ngo W., Gu B., Zhang Y., Nichols J.J. (2018). An imaging system integrating optical coherence tomography and interferometry for in vivo measurement of the thickness and dynamics of the tear film. Biomed. Eng. Online.

[16] Mastropasqua L., Nubile M., Lanzini M., Calienno R., Dua H.S. (2019). In vivo microscopic and optical coherence tomography classification of neurotrophic keratopathy. J. Cell. Physiol..

[17] Bata A.M., Witkowska K.J., Wozniak P.A., Fondi K., Schmidinger G., Pircher N., Szegedi S., Aranha Dos Santos V., Pantalon A., Werkmeister R.M. (2016). Effect of a Matrix Therapy Agent on Corneal Epithelial Healing After Standard Collagen Cross-linking in Patients With Keratoconus: A Randomized Clinical Trial. JAMA Ophthalmol..

[18] Sharma N., Singhal D., Maharana P.K., Agarwal T., Sinha R., Satpathy G., Singh Bageshwar L.M., Titiyal J.S. (2018). Spectral Domain Anterior Segment Optical Coherence Tomography in Fungal Keratitis. Cornea.

[19] Soliman W., Nassr M.A., Abdelazeem K., Al-Hussaini A.K. (2019). Appearance of herpes simplex keratitis on anterior segment optical coherence tomography. Int. Ophthalmol..

[20] Oliveira M.A., Rosa A., Soares M., Gil J., Costa E., Quadrado M.J., Murta J. (2020). Anterior Segment Optical Coherence Tomography in the Early Management of Microbial Keratitis: A Cross-Sectional Study. Acta Med. Port..

[21] Werkmeister R.M., Sapeta S., Schmidl D., Garhöfer G., Schmidinger G., Aranha Dos Santos V., Aschinger G.C., Baumgartner I., Pircher N., Schwarzhans F. (2017). Ultrahigh-resolution OCT imaging of the human cornea. Biomed. Opt. Express.

[22] Cui L., Wang J., Perez V.L., Shen M., Yuan Y., Wang M.R. (2012). Visualization of the precorneal tear film using ultrahigh resolution optical coherence tomography in dry eye. Eye Contact Lens.

[23] Gagliano C., Papa V., Amato R., Malaguarnera G., Avitabile T. (2018). Measurement of the Retention Time of Different Ophthalmic Formulations with Ultrahigh-Resolution Optical Coherence Tomography. Curr. Eye Res..

[24] Schmidl D., Witkowska K.J., Kaya S., Baar C., Faatz H., Nepp J., Unterhuber A., Werkmeister R.M., Garhofer G., Schmetterer L. (2015). The association between subjective and objective parameters for the assessment of dry-eye syndrome. Investig. Ophthalmol. Vis. Sci..

[25] Kaya S., Schmidl D., Schmetterer L., Witkowska K.J., Unterhuber A., Aranha Dos Santos V., Baar C., Garhöfer G., Werkmeister R.M. (2015). Effect of hyaluronic acid on tear film thickness as assessed with ultra-high resolution optical coherence tomography. Acta Ophthalmol..

[26] King-Smith P.E., Nichols J.J., Nichols K.K., Fink B.A., Braun R.J. (2008). Contributions of evaporation and other mechanisms to tear film thinning and break-up. Optom. Vis. Sci..

[27] Nichols J.J., Mitchell G.L., King-Smith P.E. (2005). Thinning rate of the precorneal and prelens tear films. Investig. Ophthalmol. Vis. Sci..

[28] Szegedi S., Scheschy U., Schmidl D., Aranha Dos Santos V., Stegmann H., Adzhemian N., Fondi K., Bata A.M., Werkmeister R.M., Couderc C. (2018). Effect of Single Instillation of Two Hyaluronic Acid-Based Topical Lubricants on Tear Film Thickness in Patients with Dry Eye Syndrome. J. Ocul. Pharmacol. Ther..

[29] Wozniak P.A., Schmidl D., Bata A.M., Fondi K., Witkowska K.J., Aranha Dos Santos V., Baar C., Room K.I., Nepp J., Baumgartner I. (2017). Effect of different lubricant eye gels on tear film thickness as measured with ultrahigh-resolution optical coherence tomography. Acta Ophthalmol..

[30] Schmidl D., Schmetterer L., Witkowska K.J., Unterhuber A., dos Santos V.A., Kaya S., Nepp J., Baar C., Rosner P., Werkmeister R.M. (2015). Tear film thickness after treatment with artificial tears in patients with moderate dry eye disease. Cornea.

[31] Schmidl D., Bata A.M., Szegedi S., Aranha Dos Santos V., Stegmann H., Fondi K., Krösser S., Werkmeister R.M., Schmetterer L., Garhöfer G. (2020). Influence of Perfluorohexyloctane Eye Drops on Tear Film Thickness in Patients with Mild to Moderate Dry Eye Disease: A Randomized Controlled Clinical Trial. J. Ocul. Pharmacol. Ther..

[32] Kallab M., Szegedi S., Hommer N., Stegmann H., Kaya S., Werkmeister R.M., Schmidl D., Schmetterer L., Garhöfer G. (2020). Topical Low Dose Preservative-Free Hydrocortisone Reduces Signs and Symptoms in Patients with Chronic Dry Eye: A Randomized Clinical Trial. Adv. Ther..

[33] Schmidl D., Werkmeister R., Kaya S., Unterhuber A., Witkowska K.J., Baumgartner R., Höller S., O’Rourke M., Peterson W., Wolter A. (2017). A Controlled, Randomized Double-Blind Study to Evaluate the Safety and Efficacy of Chitosan-N-Acetylcysteine for the Treatment of Dry Eye Syndrome. J. Ocul. Pharmacol. Ther..

[34] Huang J., Hindman H.B., Rolland J.P. (2016). In vivo thickness dynamics measurement of tear film lipid and aqueous layers with optical coherence tomography and maximum-likelihood estimation. Opt. Lett..

[35] Dos Santos V.A., Schmetterer L., Triggs G.J., Leitgeb R.A., Gröschl M., Messner A., Schmidl D., Garhofer G., Aschinger G., Werkmeister R.M. (2016). Super-resolved thickness maps of thin film phantoms and in vivo visualization of tear film lipid layer using OCT. Biomed. Opt. Express.

[36] Stegmann H., Aranha Dos Santos V., Messner A., Unterhuber A., Schmidl D., Garhöfer G., Schmetterer L., Werkmeister R.M. (2019). Automatic assessment of tear film and tear meniscus parameters in healthy subjects using ultrahigh-resolution optical coherence tomography. Biomed. Opt. Express.

[37] Shen M., Li J., Wang J., Ma H., Cai C., Tao A., Yuan Y., Lu F. (2009). Upper and Lower Tear Menisci in the Diagnosis of Dry Eye. Investig. Ophthalmol. Vis. Sci..

[38] Holly F.J. (1985). Physical chemistry of the normal and disordered tear film. Trans. Ophthalmol. Soc. UK..

[39] Imamura H., Tabuchi H., Nakakura S., Nagasato D., Baba H., Kiuchi Y. (2018). Usability and reproducibility of tear meniscus values generated via swept-source optical coherence tomography and the slit lamp with a graticule method. Int. Ophthalmol..

[40] Tittler E.H., Bujak M.C., Nguyen P., Zhang X., Li Y., Yiu S.C., Huang D. (2011). Between-grader repeatability of tear meniscus measurements using Fourier-domain OCT in patients with dry eye. Ophthalmic Surg. Lasers Imaging.

[41] Tukenmez-Dikmen N., Yildiz E.H., Imamoglu S., Turan-Vural E., Sevim M.S. (2016). Correlation of Dry Eye Workshop Dry Eye Severity Grading System With Tear Meniscus Measurement by Optical Coherence Tomography and Tear Osmolarity. Eye Contact Lens.

[42] Nguyen P., Huang D., Li Y., Sadda S.R., Ramos S., Pappuru R.R., Yiu S.C. (2012). Correlation between optical coherence tomography-derived assessments of lower tear meniscus parameters and clinical features of dry eye disease. Cornea.

[43] Altan-Yaycioglu R., Sizmaz S., Canan H., Coban-Karatas M. (2013). Optical coherence tomography for measuring the tear film meniscus: Correlation with schirmer test and tear-film breakup time. Curr. Eye Res..

[44] Chen Q., Zhang X., Cui L., Huang Q., Chen W., Ma H., Lu F. (2011). Upper and lower tear menisci in Sjögren’s syndrome dry eye. Investig. Ophthalmol. Vis. Sci..

[45] Qiu X., Gong L., Lu Y., Jin H., Robitaille M. (2012). The diagnostic significance of Fourier-domain optical coherence tomography in Sjögren syndrome, aqueous tear deficiency and lipid tear deficiency patients. Acta Ophthalmol..

[46] Carracedo G., Pastrana C., Serramito M., Rodriguez-Pomar C. (2019). Evaluation of tear meniscus by optical coherence tomography after different sodium hyaluronate eyedrops instillation. Acta Ophthalmol..

[47] Wang Y., Zhuang H., Xu J., Wang X., Jiang C., Sun X. (2010). Dynamic changes in the lower tear meniscus after instillation of artificial tears. Cornea.

[48] Bujak M.C., Yiu S., Zhang X., Li Y., Huang D. (2011). Serial measurement of tear meniscus by FD-OCT after instillation of artificial tears in patients with dry eyes. Ophthalmic Surg. Lasers Imaging.

[49] Bandlitz S., Purslow C., Murphy P.J., Pult H. (2019). Lid-parallel conjunctival fold (LIPCOF) morphology imaged by optical coherence tomography and its relationship to LIPCOF grade. Contact Lens Anterior Eye.

[50] Veres A., Tapaszto B., Kosina-Hagyo K., Somfai G.M., Nemeth J. (2011). Imaging lid-parallel conjunctival folds with OCT and comparing its grading with the slit lamp classification in dry eye patients and normal subjects. Investig. Ophthalmol. Vis. Sci..

[51] Kashkouli M.B., Abtahi M.B., Shahrzad S. (2015). Re: Optical Coherence Tomography Imaging of the Proximal Lacrimal System. Orbit.

[52] Timlin H.M., Keane P.A., Rose G.E., Ezra D.G. (2018). Characterizing the Occluded Lacrimal Punctum Using Anterior Segment Optical Coherence Tomography. Ophthalmic Plast. Reconstr. Surg..

[53] Timlin H.M., Keane P.A., Rose G.E., Ezra D.G. (2017). The Application of Infrared Imaging and Optical Coherence Tomography of the Lacrimal Punctum in Patients Undergoing Punctoplasty for Epiphora. Ophthalmology.

[54] Wawrzynski J.R., Smith J., Sharma A., Saleh G.M. (2014). Optical coherence tomography imaging of the proximal lacrimal system. Orbit.

[55] Bizheva K., Lee P., Sorbara L., Hutchings N., Simpson T. (2010). In vivo volumetric imaging of the human upper eyelid with ultrahigh-resolution optical coherence tomography. J. Biomed. Opt..

[56] Hwang H.S., Shin J.G., Lee B.H., Eom T.J., Joo C.K. (2013). In Vivo 3D Meibography of the Human Eyelid Using Real Time Imaging Fourier-Domain OCT. PLoS ONE.

[57] Hwang H.S., Park C.W., Joo C.K. (2013). Novel noncontact meibography with anterior segment optical coherence tomography: Hosik meibography. Cornea.

[58] Liang Q., Pan Z., Zhou M., Zhang Y., Wang N., Li B., Baudouin C., Labbe A. (2015). Evaluation of Optical Coherence Tomography Meibography in Patients With Obstructive Meibomian Gland Dysfunction. Cornea.

[59] Niederer R.L., McGhee C.N. (2010). Clinical in vivo confocal microscopy of the human cornea in health and disease. Prog. Retin. Eye Res..

[60] Erie J.C., McLaren J.W., Patel S.V. (2009). Confocal microscopy in ophthalmology. Am. J. Ophthalmol..

[61] Wang Y.E., Tepelus T.C., Vickers L.A., Baghdasaryan E., Gui W., Huang P., Irvine J.A., Sadda S., Hsu H.Y., Lee O.L. (2019). Role of in vivo confocal microscopy in the diagnosis of infectious keratitis. Int. Ophthalmol..

[62] Xiang J., Le Q., Li Y., Xu J. (2015). In vivo confocal microscopy of early corneal epithelial recovery in patients with chemical injury. Eye.

[63] Matsumoto Y., Ibrahim O.M.A. (2018). Application of In Vivo Confocal Microscopy in Dry Eye Disease. Investig. Ophthalmol. Vis. Sci..

[64] Iaccheri B., Torroni G., Cagini C., Fiore T., Cerquaglia A., Lupidi M., Cillino S., Dua H.S. (2017). Corneal confocal scanning laser microscopy in patients with dry eye disease treated with topical cyclosporine. Eye.

[65] Belmonte C., Nichols J.J., Cox S.M., Brock J.A., Begley C.G., Bereiter D.A., Dartt D.A., Galor A., Hamrah P., Ivanusic J.J. (2017). TFOS DEWS II pain and sensation report. Ocul. Surf..

[66] Al-Aqaba M.A., Dhillon V.K., Mohammed I., Said D.G., Dua H.S. (2019). Corneal nerves in health and disease. Prog. Retin. Eye Res..

[67] Liu Y., Chou Y., Dong X., Liu Z., Jiang X., Hao R., Li X. (2019). Corneal Subbasal Nerve Analysis Using In Vivo Confocal Microscopy in Patients With Dry Eye: Analysis and Clinical Correlations. Cornea.

[68] Labbé A., Alalwani H., Van Went C., Brasnu E., Georgescu D., Baudouin C. (2012). The relationship between subbasal nerve morphology and corneal sensation in ocular surface disease. Investig. Ophthalmol. Vis. Sci..

[69] Levy O., Labbé A., Borderie V., Hamiche T., Dupas B., Laroche L., Baudouin C., Bouheraoua N. (2017). Increased corneal sub-basal nerve density in patients with Sjögren syndrome treated with topical cyclosporine A. Clin. Exp. Ophthalmol..

[70] Colorado L.H., Alzahrani Y., Pritchard N., Efron N. (2016). Assessment of conjunctival goblet cell density using laser scanning confocal microscopy versus impression cytology. Contact Lens Anterior Eye.

[71] Hong J., Zhu W., Zhuang H., Xu J., Sun X., Le Q., Li G., Wang Y. (2010). In vivo confocal microscopy of conjunctival goblet cells in patients with Sjogren’s syndrome dry eye. Br. J. Ophthalmol..

[72] Machetta F., Fea A.M., Actis A.G., de Sanctis U., Dalmasso P., Grignolo F.M. (2014). In vivo confocal microscopic evaluation of corneal langerhans cells in dry eye patients. Open Ophthalmol. J..

[73] Wakamatsu T.H., Sato E.A., Matsumoto Y., Ibrahim O.M., Dogru M., Kaido M., Ishida R., Tsubota K. (2010). Conjunctival in vivo confocal scanning laser microscopy in patients with Sjögren syndrome. Investig. Ophthalmol. Vis. Sci..

[74] Randon M., Aragno V., Abbas R., Liang H., Labbé A., Baudouin C. (2019). In vivo confocal microscopy classification in the diagnosis of meibomian gland dysfunction. Eye.

[75] Zhao H., Chen J.Y., Wang Y.Q., Lin Z.R., Wang S. (2016). In vivo Confocal Microscopy Evaluation of Meibomian Gland Dysfunction in Dry Eye Patients with Different Symptoms. Chin. Med. J..

[76] Tomlinson A., Bron A.J., Korb D.R., Amano S., Paugh J.R., Pearce E.I., Yee R., Yokoi N., Arita R., Dogru M. (2011). The international workshop on meibomian gland dysfunction: Report of the diagnosis subcommittee. Investig. Ophthalmol. Vis. Sci..

[77] Arita R. (2018). Meibography: A Japanese Perspective. Investig. Ophthalmol. Vis. Sci..

[78] Yokoi N., Komuro A., Yamada H., Maruyama K., Kinoshita S. (2007). A newly developed video-meibography system featuring a newly designed probe. JPN J. Ophthalmol..

[79] Arita R., Fukuoka S., Morishige N. (2017). New Insights Into the Lipid Layer of the Tear Film and Meibomian Glands. Eye Contact Lens.

[80] Finis D., Ackermann P., Pischel N., König C., Hayajneh J., Borrelli M., Schrader S., Geerling G. (2015). Evaluation of Meibomian Gland Dysfunction and Local Distribution of Meibomian Gland Atrophy by Non-contact Infrared Meibography. Curr. Eye Res..

[81] AlDarrab A., Alrajeh M., Alsuhaibani A.H. (2017). Meibography for eyes with posterior blepharitis. Saudi J. Ophthalmol..

[82] Arita R., Suehiro J., Haraguchi T., Maeda S., Maeda K., Tokoro H., Amano S. (2013). Topical diquafosol for patients with obstructive meibomian gland dysfunction. Br. J. Ophthalmol..

[83] Maskin S.L., Testa W.R. (2018). Growth of meibomian gland tissue after intraductal meibomian gland probing in patients with obstructive meibomian gland dysfunction. Br. J. Ophthalmol..

[84] Arita R., Itoh K., Maeda S., Maeda K., Furuta A., Tomidokoro A., Aihara M., Amano S. (2012). Effects of long-term topical anti-glaucoma medications on meibomian glands. Graefes Arch. Clin. Exp. Ophthalmol..

[85] Düzgün E., Özkur E. (2020). The effect of oral isotretinoin therapy on meibomian gland morphology and dry eye tests. J. Dermatol. Treat..

[86] Maskin S.L., Alluri S. (2020). Meibography guided intraductal meibomian gland probing using real-time infrared video feed. Br. J. Ophthalmol..

[87] Blackie C.A., Solomon J.D., Scaffidi R.C., Greiner J.V., Lemp M.A., Korb D.R. (2009). The relationship between dry eye symptoms and lipid layer thickness. Cornea.

[88] Markoulli M., Duong T.B., Lin M., Papas E. (2018). Imaging the Tear Film: A Comparison Between the Subjective Keeler Tearscope-Plus™ and the Objective Oculus^®^ Keratograph 5M and LipiView^®^ Interferometer. Curr. Eye Res..

[89] Guillon M., Styles E., Guillon J.P., Maïssa C. (1997). Preocular tear film characteristics of nonwearers and soft contact lens wearers. Optom. Vis. Sci..

[90] Eom Y., Lee J.S., Kang S.Y., Kim H.M., Song J.S. (2013). Correlation between quantitative measurements of tear film lipid layer thickness and meibomian gland loss in patients with obstructive meibomian gland dysfunction and normal controls. Am. J. Ophthalmol..

[91] Knop E., Knop N., Millar T., Obata H., Sullivan D.A. (2011). The international workshop on meibomian gland dysfunction: Report of the subcommittee on anatomy, physiology, and pathophysiology of the meibomian gland. Investig. Ophthalmol. Vis. Sci..

[92] Suzuki S., Goto E., Dogru M., Asano-Kato N., Matsumoto Y., Hara Y., Fujishima H., Tsubota K. (2006). Tear film lipid layer alterations in allergic conjunctivitis. Cornea.

[93] Fogt J.S., Kowalski M.J., King-Smith P.E., Epitropolous A.T., Hendershot A.J., Lembach C., Maszczak J.P., Jones-Jordan L.A., Barr J.T. (2016). Tear lipid layer thickness with eye drops in meibomian gland dysfunction. Clin. Ophthalmol..

[94] Fukuoka S., Arita R. (2017). Increase in tear film lipid layer thickness after instillation of 3% diquafosol ophthalmic solution in healthy human eyes. Ocul. Surf..

[95] Korb D.R., Scaffidi R.C., Greiner J.V., Kenyon K.R., Herman J.P., Blackie C.A., Glonek T., Case C.L., Finnemore V.M., Douglass T. (2005). The effect of two novel lubricant eye drops on tear film lipid layer thickness in subjects with dry eye symptoms. Optom. Vis. Sci..

[96] Li Y., Sang X., Yang L., Wang X.R., Liu J.H., He X.J., Liu Y., Lu X.H., Wang Z.C. (2018). Low concentration of sodium hyaluronate temporarily elevates the tear film lipid layer thickness in dry eye patients with lipid deficiency. Int. J. Ophthalmol..

[97] Goto E., Dogru M., Fukagawa K., Uchino M., Matsumoto Y., Saiki M., Tsubota K. (2006). Successful tear lipid layer treatment for refractory dry eye in office workers by low-dose lipid application on the full-length eyelid margin. Am. J. Ophthalmol..

[98] Lee S.M., Lee J.E., Kim S.I., Jung J.H., Shin J. (2019). Effect of topical glaucoma medication on tear lipid layer thickness in patients with unilateral glaucoma. Indian J. Ophthalmol..

[99] Kim J.S., Lee H., Choi S., Kim E.K., Seo K.Y., Kim T.I. (2018). Assessment of the Tear Film Lipid Layer Thickness after Cataract Surgery. Semin. Ophthalmol..

[100] Arita R., Yabusaki K., Hirono T., Yamauchi T., Ichihashi T., Fukuoka S., Morishige N. (2019). Automated Measurement of Tear Meniscus Height with the Kowa DR-1α Tear Interferometer in Both Healthy Subjects and Dry Eye Patients. Investig. Ophthalmol. Vis. Sci..

[101] Johnson M.E., Murphy P.J. (2005). The Effect of instilled fluorescein solution volume on the values and repeatability of TBUT measurements. Cornea.

[102] Liu Z., Pflugfelder S.C. (1999). Corneal surface regularity and the effect of artificial tears in aqueous tear deficiency. Ophthalmology.

[103] Goto T., Zheng X., Klyce S.D., Kataoka H., Uno T., Karon M., Tatematsu Y., Bessyo T., Tsubota K., Ohashi Y. (2003). A new method for tear film stability analysis using videokeratography. Am. J. Ophthalmol..

[104] Goto T., Zheng X., Okamoto S., Ohashi Y. (2004). Tear film stability analysis system: Introducing a new application for videokeratography. Cornea.

[105] Kojima T., Ishida R., Dogru M., Goto E., Takano Y., Matsumoto Y., Kaido M., Ohashi Y., Tsubota K. (2004). A new noninvasive tear stability analysis system for the assessment of dry eyes. Investig. Ophthalmol. Vis. Sci..

[106] Gumus K., Crockett C.H., Rao K., Yeu E., Weikert M.P., Shirayama M., Hada S., Pflugfelder S.C. (2011). Noninvasive assessment of tear stability with the tear stability analysis system in tear dysfunction patients. Investig. Ophthalmol. Vis. Sci..

[107] Best N., Drury L., Wolffsohn J.S. (2012). Clinical evaluation of the Oculus Keratograph. Contact Lens Anterior Eye.

[108] Hong J., Sun X., Wei A., Cui X., Li Y., Qian T., Wang W., Xu J. (2013). Assessment of tear film stability in dry eye with a newly developed keratograph. Cornea.

[109] Alonso-Caneiro D., Iskander D.R., Collins M.J. (2009). Tear film surface quality with soft contact lenses using dynamic-area high-speed videokeratoscopy. Eye Contact Lens.

[110] Iskander D.R., Collins M.J. (2005). Applications of high-speed videokeratoscopy. Clin. Exp. Optom.

[111] Downie L.E. (2015). Automated Tear Film Surface Quality Breakup Time as a Novel Clinical Marker for Tear Hyperosmolarity in Dry Eye Disease. Investig. Ophthalmol. Vis. Sci..

[112] Mengher L.S., Bron A.J., Tonge S.R., Gilbert D.J. (1985). A non-invasive instrument for clinical assessment of the pre-corneal tear film stability. Curr. Eye Res..

[113] Ozulken K., Aksoy Aydemir G., Tekin K., Mumcuoğlu T. (2020). Correlation of Non-invasive Tear Break-Up Time with Tear Osmolarity and Other Invasive Tear Function Tests. Semin. Ophthalmol..

[114] Bhandari V., Reddy J.K., Relekar K., Ingawale A., Shah N. (2016). Non-invasive assessment of tear film stability with a novel corneal topographer in Indian subjects. Int. Ophthalmol..

[115] Ngo W., Srinivasan S., Houtman D., Jones L. (2017). The relief of dry eye signs and symptoms using a combination of lubricants, lid hygiene and ocular nutraceuticals. J. Optom..

[116] Konieczka K., Schoetzau A., Koch S., Hauenstein D., Flammer J. (2018). Cornea Thermography: Optimal Evaluation of the Outcome and the Resulting Reproducibility. Transl. Vis. Sci. Technol..

[117] Chan T.C.Y., Wan K.H., Shih K.C., Jhanji V. (2018). Advances in dry eye imaging: The present and beyond. Br. J. Ophthalmol..

[118] Craig J.P., Singh I., Tomlinson A., Morgan P.B., Efron N. (2000). The role of tear physiology in ocular surface temperature. Eye.

[119] Szczesna D.H., Alonso-Caneiro D., Iskander D.R., Read S.A., Collins M.J. (2011). Predicting dry eye using noninvasive techniques of tear film surface assessment. Investig. Ophthalmol. Vis. Sci..

[120] Klamann M.K., Maier A.K., Gonnermann J., Klein J.P., Pleyer U. (2012). Measurement of dynamic ocular surface temperature in healthy subjects using a new thermography device. Curr. Eye Res..

[121] Kamao T., Yamaguchi M., Kawasaki S., Mizoue S., Shiraishi A., Ohashi Y. (2011). Screening for dry eye with newly developed ocular surface thermographer. Am. J. Ophthalmol..

[122] Kawali A.A. (2013). Thermography in ocular inflammation. Indian J. Radiol. Imaging.

[123] Morgan P.B., Tullo A.B., Efron N. (1995). Infrared thermography of the tear film in dry eye. Eye.

[124] Fujishima H., Toda I., Yamada M., Sato N., Tsubota K. (1996). Corneal temperature in patients with dry eye evaluated by infrared radiation thermometry. Br. J. Ophthalmol..

[125] Azharuddin M., Bera S.K., Datta H., Dasgupta A.K. (2014). Thermal fluctuation based study of aqueous deficient dry eyes by non-invasive thermal imaging. Exp. Eye Res..

[126] Su T.Y., Ho W.T., Lu C.Y., Chang S.W., Chiang H.K. (2015). Correlations among ocular surface temperature difference value, the tear meniscus height, Schirmer’s test and fluorescein tear film break up time. Br. J. Ophthalmol..

[127] Su T.Y., Chang S.W., Yang C.J., Chiang H.K. (2014). Direct observation and validation of fluorescein tear film break-up patterns by using a dual thermal-fluorescent imaging system. Biomed. Opt. Express.

[128] Li W., Graham A.D., Selvin S., Lin M.C. (2015). Ocular Surface Cooling Corresponds to Tear Film Thinning and Breakup. Optom. Vis. Sci..

[129] Abreau K., Callan C., Kottaiyan R., Zhang A., Yoon G., Aquavella J.V., Zavislan J., Hindman H.B. (2016). Temperatures of the Ocular Surface, Lid, and Periorbital Regions of Sjogren’s, Evaporative, and Aqueous-Deficient Dry Eyes Relative to Normals. Ocul. Surf..

[130] Matteoli S., Favuzza E., Mazzantini L., Aragona P., Cappelli S., Corvi A., Mencucci R. (2017). Ocular surface temperature in patients with evaporative and aqueous-deficient dry eyes: A thermographic approach. Physiol. Meas..

[131] Segev F., Geffen N., Galor A., Cohen Y., Gefen R., Belkin A., Arieli Y., Epshtein S., Oren A., Harris A. (2020). Dynamic assessment of the tear film muco-aqueous and lipid layers using a novel tear film imager (TFI). Br. J. Ophthalmol..

[132] Cohen Y., Trokel S., Arieli Y., Epshtien S., Gefen R., Harris A. (2020). Mapping the Lipid Layer of the Human Tear Film. Cornea.

[133] Bron A.J., de Paiva C.S., Chauhan S.K., Bonini S., Gabison E.E., Jain S., Knop E., Markoulli M., Ogawa Y., Perez V. (2017). TFOS DEWS II pathophysiology report. Ocul. Surf..

[134] Wang M.T.M., Tien L., Han A., Lee J.M., Kim D., Markoulli M., Craig J.P. (2018). Impact of blinking on ocular surface and tear film parameters. Ocul. Surf..

[135] Chou Y.B., Fan N.W., Lin P.Y. (2019). Value of lipid layer thickness and blinking pattern in approaching patients with dry eye symptoms. Can. J. Ophthalmol..

[136] Jie Y., Sella R., Feng J., Gomez M.L., Afshari N.A. (2019). Evaluation of incomplete blinking as a measurement of dry eye disease. Ocul. Surf..

[137] Pult H., Riede-Pult B.H., Murphy P.J. (2013). The relation between blinking and conjunctival folds and dry eye symptoms. Optom. Vis. Sci..

[138] Su Y., Liang Q., Su G., Wang N., Baudouin C., Labbé A. (2018). Spontaneous Eye Blink Patterns in Dry Eye: Clinical Correlations. Investig. Ophthalmol. Vis. Sci..

[139] Johnston P.R., Rodriguez J., Lane K.J., Ousler G., Abelson M.B. (2013). The interblink interval in normal and dry eye subjects. Clin. Ophthalmol..

[140] Tsubota K., Hata S., Okusawa Y., Egami F., Ohtsuki T., Nakamori K. (1996). Quantitative videographic analysis of blinking in normal subjects and patients with dry eye. Arch. Ophthalmol..

[141] Ang C.K., Mohidin N., Chung K.M. (2014). Effects of wink glass on blink rate, nibut and ocular surface symptoms during visual display unit use. Curr. Eye Res..

[142] Schlote T., Kadner G., Freudenthaler N. (2004). Marked reduction and distinct patterns of eye blinking in patients with moderately dry eyes during video display terminal use. Graefes Arch. Clin. Exp. Ophthalmol..

[143] Maeda N. (2009). Clinical applications of wavefront aberrometry—A review. Clin. Exp. Ophthalmol..

[144] Koh S., Tung C., Aquavella J., Yadav R., Zavislan J., Yoon G. (2010). Simultaneous measurement of tear film dynamics using wavefront sensor and optical coherence tomography. Investig. Ophthalmol. Vis. Sci..

[145] Denoyer A., Rabut G., Baudouin C. (2012). Tear film aberration dynamics and vision-related quality of life in patients with dry eye disease. Ophthalmology.

[146] Deschamps N., Ricaud X., Rabut G., Labbé A., Baudouin C., Denoyer A. (2013). The impact of dry eye disease on visual performance while driving. Am. J. Ophthalmol..

[147] Lekhanont K., Chuckpaiwong V., Vongthongsri A., Sangiampornpanit T. (2014). Effects of sodium hyaluronate on wavefront aberrations in dry eye patients. Optom. Vis. Sci..

[148] Koh S., Inoue Y., Sugmimoto T., Maeda N., Nishida K. (2013). Effect of rebamipide ophthalmic suspension on optical quality in the short break-up time type of dry eye. Cornea.

[149] Schmidt-Erfurth U., Chong V., Loewenstein A., Larsen M., Souied E., Schlingemann R., Eldem B., Monés J., Richard G., Bandello F. (2014). Guidelines for the management of neovascular age-related macular degeneration by the European Society of Retina Specialists (EURETINA). Br. J. Ophthalmol..

[150] Cicinelli M.V., Cavalleri M., Brambati M., Lattanzio R., Bandello F. (2019). New imaging systems in diabetic retinopathy. Acta Diabetol..

[151] Bussel I.I., Wollstein G., Schuman J.S. (2014). OCT for glaucoma diagnosis, screening and detection of glaucoma progression. Br. J. Ophthalmol..

[152] McDonald M., Patel D.A., Keith M.S., Snedecor S.J. (2016). Economic and Humanistic Burden of Dry Eye Disease in Europe, North America, and Asia: A Systematic Literature Review. Ocul. Surf..

[153] Ting D.S.W., Pasquale L.R., Peng L., Campbell J.P., Lee A.Y., Raman R., Tan G.S.W., Schmetterer L., Keane P.A., Wong T.Y. (2019). Artificial intelligence and deep learning in ophthalmology. Br. J. Ophthalmol..

[154] Tabuchi H., Masumoto H. (2020). Objective evaluation of allergic conjunctival disease (with a focus on the application of artificial intelligence technology). Allergol. Int..

[155] Hao H., Zhao Y., Fu H., Shang Q., Li F., Zhang X., Liu J. Anterior Chamber Angles Classification in Anterior Segment OCT Images via Multi-Scale Regions Convolutional Neural Networks. Proceedings of the 2019 41st Annual International Conference of the IEEE Engineering in Medicine and Biology Society.

[156] Treder M., Lauermann J.L., Alnawaiseh M., Eter N. (2019). Using Deep Learning in Automated Detection of Graft Detachment in Descemet Membrane Endothelial Keratoplasty: A Pilot Study. Cornea.

